# Morphology-Based Identification of *Bemisia tabaci* Cryptic Species Puparia via Embedded Group-Contrast Convolution Neural Network Analysis

**DOI:** 10.1093/sysbio/syab098

**Published:** 2021-12-24

**Authors:** Norman MacLeod, Roy J Canty, Andrew Polaszek

**Affiliations:** School of Earth Sciences and Engineering, Zhu Gongshan Building, 163 Xianlin Avenue, Nanjing, 210023 Jiangsu, China; Department of Entomology, Staatliches Museum für Naturkunde, Rosenstein 1, 70191 Stuttgart, Germany; Department of Life Sciences, Natural History Museum, Cromwell Road, SW7 5BD London, UK; Department of Life Sciences, Natural History Museum, Cromwell Road, SW7 5BD London, UK

## Abstract

The *Bemisia tabaci* species complex is a group of tropical–subtropical hemipterans, some species of which have achieved global distribution over the past 150 years. Several species are regarded currently as among the world’s most pernicious agricultural pests, causing a variety of damage types via direct feeding and plant-disease transmission. Long considered a single variable species, genetic, molecular and reproductive compatibility analyses have revealed that this “species” is actually a complex of between 24 and 48 morphologically cryptic species. However, determinations of which populations represent distinct species have been hampered by a failure to integrate genetic/molecular and morphological species–diagnoses. This, in turn, has limited the success of outbreak-control and eradication programs. Previous morphological investigations, based on traditional and geometric morphometric procedures, have had limited success in identifying genetic/molecular species from patterns of morphological variation in puparia. As an alternative, our investigation focused on exploring the use of a deep-learning convolution neural network (CNN) trained on puparial images and based on an embedded, group-contrast training protocol as a means of searching for consistent differences in puparial morphology. Fifteen molecular species were selected for analysis, all of which had been identified via DNA barcoding and confirmed using more extensive molecular characterizations and crossing experiments. Results demonstrate that all 15 species can be discriminated successfully based on differences in puparium morphology alone. This level of discrimination was achieved for laboratory populations reared on both hairy-leaved and glabrous-leaved host plants. Moreover, cross-tabulation tests confirmed the generality and stability of the CNN discriminant system trained on both ecophenotypic variants. The ability to identify *B. tabaci* species quickly and accurately from puparial images has the potential to address many long-standing problems in *B. tabaci* taxonomy and systematics as well as playing a vital role in ongoing pest-management efforts. [Aleyrodidae; entomology; Hemiptera; machine learning; morphometrics; pest control; systematics; taxonomy; whiteflies.]

The whitefly *Bemisia tabaci* (Gennadius) (Hemiptera: Aleyrodidae) is a widespread cryptic species complex, with the species “MEAM1” and “MED” (formerly biotypes B and B2, and biotypes Q, J, and L, respectively) having acquired a cosmopolitan distribution (Hu et al. 2011). This group includes species that are among the world’s most destructive horticultural pests (Tay et al. 2017; Vyskočilová et al. 2018). Species show variation in puparial, as well as adult, morphology, of which the former is often correlated with leaf characteristics such as degree of hairiness (Mound 1963).

Initially, the cryptic nature of morphological variation in the *B. tabaci* complex created taxonomic confusion, leading to large-scale species-level synonymy (e.g., Russell 1957; Mound 1963). There are, however, several, often subtle, interspecific differences that have indicated *B. tabaci* might be a cryptic species complex, including differences in insecticide resistance (Hussain et al. 2019), host preference, and range (Bellows et al. 1994; Vyskočilová et al. 2019), parasitoid (Liu et al. 2016) associated endosymbiont (Marubayashi et al. 2014) faunas, and virus transmissions (Pan et al. 2018; Hussain et al. 2019; Chi et al. 2020; Fiallo-Olivé et al. 2020).

*Bemisia tabaci* infestations often lead to widespread and extensive crop damage via direct feeding, plant physiological disorders, honeydew contamination with associated fungal growth and, most importantly, the transmission of viral diseases (Oliveira et al. 2001; Navas-Castillo et al. 2011). Induced damage can effect the quality and/or quantity of agricultural produce severely. In addition, many species have attracted considerable attention because of their ability to evolve pesticide resistance (e.g., Basit 2019), high-dispersal abilities, and extremely polyphagous diets, though others appear to be relatively host-specific (Vyskočilová et al. 2018). As a result, a pressing need exists to keep *B. tabaci* infestations of agricultural crops under control, with biocontrol being the preferred method (Jazzar and Hammad 2004). For biocontrol to be successful, however, accurate species identifications are required (Rosen 1986; Schauff and La Salle 1998; Peixoto et al. 2018).

According to the latest species-diversity assessments, based either on DNA-sequencing methods on mtCO1 in isolation (Dinsdale et al. 2010) or in combination with other data (Kanakala and Ghanim 2019; Vyskočilová et al. 2019), the *B. tabaci* complex may contain as many as 48 species (Kanakala and Ghanim 2019; Vyskočilová et al. 2019). Mostly, crossing experiments have demonstrated partial, or complete, reproductive isolation between populations identified as separate species a priori by CO1 barcoding (De Barro and Hart 2000; Liu et al. 2012; Qin et al. 2015; Vyskočilová et al. 2019). Nonetheless, despite clear molecular evidence that *B. tabaci* is a species complex, some recent publications continue to refer to, and treat, it as a single species (e.g., Becht et al. 2019; Ueda et al. 2019). While acknowledging *B. tabaci* as a species complex, others refer to it ambiguously (e.g., Hamada et al. 2019) or inconsistently (e.g., Kliot et al. 2019). The current lack of precision in making routine species-level identifications has created difficulties in the reproduction of research results in addition to compromising the effectiveness of control procedures, both of which are becoming common issues in many entomological research contexts (e.g., Packer et al. 2018).

Recently, several authors have called for a more integrative approach to the assessment of species taxonomy, involving several complimentary disciplines (Dayrat 2005; Tan et al. 2009; Schlick-Steiner et al. 2010). Considering that most insect species are diagnosed by their morphological characteristics, Tan et al. (2009) have argued that morphology should be considered in all entomological studies. Furthermore, though morphologically distinct, *B. atriplex* still clusters genetically with some *B. tabaci* lineages (Lee et al. 2013; Mugerwa et al. 2018). These results emphasize the need to determine whether consistent interspecific morphological characteristics are exhibited by some or all *B. tabaci*-complex species (Lee et al. 2013).

Whitefly (Aleyrodidae) taxon delimitation, both currently and historically, has been based almost entirely on the morphology of the final instar larva, the so-called puparium (Gill 1990; Bellows et al. 1994; Hodges and Evans 2005). Puparia are cleared and mounted on slides for microscopic examination, typically using enhanced lighting methods (e.g., phase-contrast; Nomarski differential interference contrast) at magnifications of from $40\times $ to $400\times $ (Manzari and Quicke 2006). Adults have rarely been used for taxonomic study due to their extremely delicate nature (shriveling completely when dead) and being covered with wax. Preliminary (unpublished) observations suggest some potential exists for species diagnosis based on adult characters, especially male genitalia. But for reasons of practicality, history and comparison with previous morphometric studies, adult characters were considered outside the scope of our investigation.

To date, a very limited range of morphometric procedures have been applied to the *B. tabaci* complex. Bethke et al. (1991) examined sexual dimorphism in rates of development, body sizes of adult and puparial instars, and adult tibia lengths in two populations, one reared on cotton and the other on poinsettia plants, using linear distance measurements. Body sizes for both puparia and adults were found to be larger in the cotton-reared population and female pupae were found to be larger than male pupae overall. Li et al. (2013) employed length and width measurements collected from four instars of six Chinese *B. tabaci* “biotypes” (all reared on cotton plants), in addition to length and width measurements of six additional characters from the fourth instar, to study interbiotype morphological distinctions. This study achieved a general puparial biotype size ranking and identified three biotypes (B, Q, and ZHJ-2) as differing from the rest in three characters. With respect to larval biotype distinctions, Li et al. (2013) concluded that exotic biotypes were significantly larger than indigenous forms. Similarly, Harish et al. (2016) undertook a principal components analysis (PCA) of 14 puparial characteristics, including total body length and width measurements, widths of the wax margins, and nine different adult characteristics, but found these insufficient to distinguish populations from different agro-ecological zones reliably. Geometric morphometric (GM) analysis has also been applied previously to *B. tabaci* puparial data with somewhat disappointing results in terms of its ability to detect population-level differences (see Chaubey and Andrew 2015).

While the Li et al. (2013), Chaubey and Andrew (2015), and Harish et al. (2016) studies all suggested close inspection of *B. tabaci* puparium morphological characters had promise in being able to achieve diagnoses of at least some genetic/molecular species, compelling empirical demonstrations of this potential eluded each of these research groups. But rather than concluding puparium morphology-based distinctions between established genetic/molecular species do not exist—as some have done—perhaps the problem has lain in the approaches used to analyze morphological data for distinctions that reflect, genetic/molecular species differences.

The use of linear distances between topologically homologous landmark points represents, by contemporary morphometric standards, a rather crude, substandard, and inherently problematic approach to the analysis of morphology insofar as such distances are simply scalar magnitudes that encode no aspect of the geometries from which they were obtained (Bookstein 1991). Nonetheless, the indistinct, and often times difficult-to-image character of *B. tabaci* puparial morphologies (see Fig. 1) precludes the application of more advanced landmark and/or semilandmark-based, morphometric data-collection procedures. Recently, machine-learning (ML) algorithms have been applied to the problem of morphological group-discrimination in which the input data are digital images of the specimens themselves (see MacLeod et al. 2005; MacLeod 2007, 2015, 2018; Ranaweera et al. 2009; Wilf et al. 2016; Valan et al. 2019; Hoyal Cuthill et al. 2019; MacLeod and Kolska-Horwitz 2020). Increasingly, ML procedures have been shown to be capable of delivering morphological group-discrimination results superior to those of even the most advanced GM-style analyses (MacLeod 2015, 2018; Hoyal Cuthill et al. 2019; MacLeod and Kolska-Horwitz 2020). Accordingly, the primary aim of this investigation was to determine whether the application of ML and computer vision-based morphological data and data-analysis methods might facilitate the identification of puparium-based morphological distinctions between members of the *B. tabaci* complex represented by a large set of currently recognized genetic species. Specific objectives of our investigation included the following.

i. Determination of whether statistically reliable morphology-based taxonomic identifications could be made from simple, transmitted-light photomicrographs of puparial specimens.
ii. Documentation of the extent to which any between-group distinctions exhibit continuous or disjunct distributions in the image feature space, the latter of which might, in principle, serve as the basis for development of a reliable and accurate approach to automating the identification of at least some *B. tabaci* species from morphological data.
iii. Evaluation to test whether any observed between-species distinctions represent consistently structured morphological differences, and are not reflections of system overtraining or sampling effects as indicated by statistical tests at appropriate significance levels.
iv. Comparison to learn whether any between-groups distinctions recognized were more prominent, less prominent, or equally prominent with regard to specimens reared on different host plant leaf-surface types owing to the well-known ecophenotypic plasticity of this taxon.

**Figure 1:**
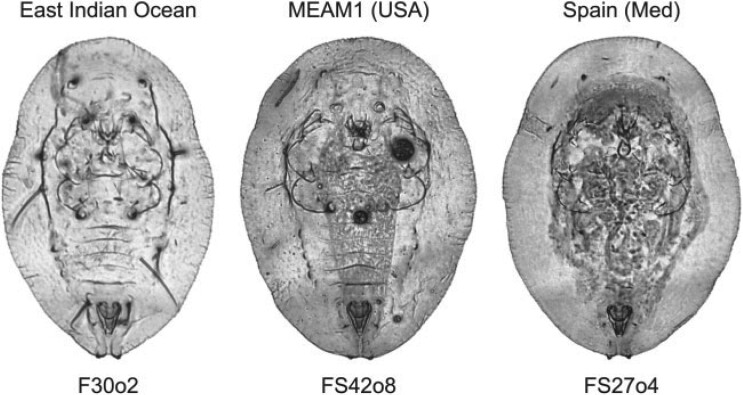
Images of three representative puparium specimens from the *Bemisia tabaci* complex illustrating variation in the level of morphological detail present in our sample. Aside from the form outlines and position of the posteriorly positioned vasiform structure there are few consistently locatable topologically homologous points of reference that could be used to represent these morphologies either accurately or comprehensively.

## Materials and Methods

### Specimen Cultures

All *B. tabaci* complex specimens used in this study were reared in cultures in the laboratories of the University of Greenwich’s Natural Resources Institute (NRI, https://www.nri.org). These cultures were separated into distinct genetic species based on the findings of Dinsdale et al. (2010), and reared under strict quarantine conditions. Due to their morphological plasticity, each species colony was further split into two or more separate colonies, and allowed to feed on either hairy or glabrous leaves. See the Supplementary Material archive available on Dryad at https://doi.org/10.5061/dryad.sqv9s4n39 for a complete description of specimen-rearing conditions and procedures.

### Specimen Collecting and Selection

Specimens were collected by taking cuttings of leaves hosting whiteflies. While in storage, specimens were selected for study. Only puparia from which no adult had emerged were selected for DNA extraction and to ensure that there would be no anomalous image data due to the presence of moulting sutures. Genomic DNA was extracted from each specimen following the protocol described by Polaszek et al. (2013). Immediately after removal from the enzyme/buffer mix, all puparia were processed for slide mounting. Subsequent to washing, the method outlined in Sirisena et al. (2013) for assembling permanent slide-mounts was followed. The only deviation from this method implemented was to forego the staining of specimens as uneven staining leads to artifacts that could affect image quality and, therefore, image-analysis results.

Only puparia showing the clearest morphology (e.g., clear vasiform orifice, clear abdominal segments, clear dorsal disc margin) in their groups and host-plant types were imaged to ensure sufficient morphological detail was present for subsequent morphological analysis. In order to assemble an initial, high-resolution image (c. $1050 \times 1550$ pixels @ 300 dpi) of each puparium’s full morphology all puparia were imaged at $20\times $ magnification. Focus stacking (Sidney 2002; Johnson 2008) was used to overcome the fact that, at this magnification, the focal depth was too shallow to obtain clear images of all internal morphological structures. This operation required collection of between 5 and 30 images, as specimens varied in thickness. Since the $20\times $ field-of-view was also unable to include the whole specimen, each specimen was subdivided into six sections. These sections were imaged individually (using focus stacking) and their boundaries stitched together in software (see Szeliski 2006) to produce a fully focused composite image of the entire specimen. These composite images were then processed to convert them to a grayscale palette using the chromatic adaptation method (effected via the Von Kries transform) with a setting of D65 being used as the cone-response white point for the original RGB images. This operation preserved the appearance of the specimens despite changes in the illumination of the three color channels. Brightness and contrast adjustments were also made (by hand) to ensure all images i) exhibited broadly comparable exposures with one another and ii) retained a degree of uniqueness which enhanced the ability of the ML algorithm to find well-structured within-groups patterns of similarity and between-groups patterns of difference. Finally, each processed image was selected and cropped to remove background pixels, copied onto a pure white-pixel image frame, and the layers merged to provide a single-layer image file. A summary of the groups and sample sizes employed in our analysis is provided in Table 1 and the image sets for both hairy leaf-reared and glabrous leaf-reared specimens included in the Supplementary Materials archive available on Dryad

**Table 1.** Sizes of *Bemisia tabacai* group samples used in this investigation

Group	Hairy	Glabrous
East-Indian Ocean	34	30
Israel (Med)	37	32
MEAM1 (Australia)	32	30
MEAM1 (Peru)	30	31
MEAM1 (USA)	32	31
Spain (Med)	24	17
Uganda (Med)	22	11
Uganda (Med-SP)	25	25
SSA1 (SG1)	21	26
SSA1 (SG2)	19	22
SSA1 (SG3)	21	24
SSA2 (Africa)	22	19
SSA2 (Spain)	20	19
SSA3	24	22
Sudan (S)	23	27
Total	386	366

### Embedded (Deep Learning) Convolution Neural Network Analysis

A “deep learning” convolution neural network (CNN) analysis, using the LeNet-5 architecture (LeCun et al. 1998, 2015), was employed to compare and analyze differences among and between genetic/molecular species directly. LeNet-5 was the CNN that sparked initial widespread interest in “deep learning” using convolution-based, multilayer artificial neural networks after it achieved 98.5$\%$ accuracy when tested on the images included in the Modified Nation Institute of Standards and Technology (MNIST) image database (see http://yann.lecun.com/exdb/mnist/).

All CNNs consist of an input layer that receives the information to be processed (in our case images of *B. tabaci* puparia) and an output layer that makes the final allocation of the processed data into one of a number of categories or classes (in our case species). Between these, a variable number of connected or hidden” layers exist that process the data by i) accepting the information from the input or previous layers, ii) evaluating this information for patterns consistent with those established by a training set of authoritatively identified images, and iii) passing these processed data on to the next layer. For our analysis we adopted the standard LeNet default of autoencoding, or “stepping down” the input image resolutions to $28 \times 28$, 8-bit, grayscale, pixel values as an initial processing step. (Note: standardization to a $28 \times 28$ pixel image is a setting that can be varied in the LeNet-5 architecture depending on the complexity of the images being analyzed).

Although LeNet-5 is but one of several advanced, gradient-descent CNN architectures for image-based automated identification applications (e.g., ResNet, VGG; see https://resources.wolframcloud.com/NeuralNetRepository), it remains one of the most efficient, best understood, and most flexible architectures available currently. LeNet-5 also has the advantage of requiring estimation of a much smaller number of trainable parameters (c. 60,000 trainable parameters) and, as a result, being more suitable for the analysis of small samples than larger, more complex deep-learning architectures. Moreover, its simple and rather straight-forward design makes visual inspection of the LeNet-5 convolution filters employed to achieve group separation a tractable proposition. Today, the LeNet-5 architecture is used commonly to illustrate the basic principles of CNN design and application to image-identification problems. We make no claim that LeNet-5 is the most advanced, or even the best, deep-learning CNN available, only that it was adequate to the needs of our investigation as judged objectively by the results we have obtained through its use. The overall structure of the LeNet-5 architecture employed is listed in Table 2 and a complete listing of the code used in the software written for this investigation is available in the Supplementary Materials archive available on Dryad.

**Table 2.** Layer structure of the LeNet-5 “deep learning” CNN employed in this investigation. Sizes refer to pixels for layers 1–7, variables for layers 8–10

Layers	Type	Parameters
		Image
1	Input	3-tensor (size: $1 \times 28 \times 28$)
2	Convolution	3-tensor (size: $10 \times 25 \times 25$)
3	Ramp	3-tensor (size: $10 \times 25 \times 25$)
4	Pooling	3-tensor (size: $10 \times 12 \times 12$)
5	Convolution	3-tensor (size: $20 \times 9 \times 9$)
6	Ramp	3-tensor (size: $20 \times 9 \times 9$)
7	Pooling	3-tensor (size: $20 \times 4 \times 4$)
8	Flatten	vector (size: 320)
9	Linear	vector (size: 2)
10	Output	vector (size: 2)

With respect to this CNN design, it is important to point out that the output-vector dimension, which is usually set to the number of groups resident in the training set, was set to 2 for our analysis so the system would output a 2D, trained feature space. This parameter setting facilitated complete illustration of the feature space into which the training set specimens were projected. Adoption of this variation, in turn, allowed the complete between-groups separation achieved by LeNet-5 training to be illustrated, thereby allowing us to realize objective iii of our investigation (see above) unambiguously. Tests using both the (more traditional) output vector setting of 15 and 2 showed no difference between identification accuracies achieved by the LeNet-5 architecture for our *B. tabaci* image sets.

**Figure 2:**
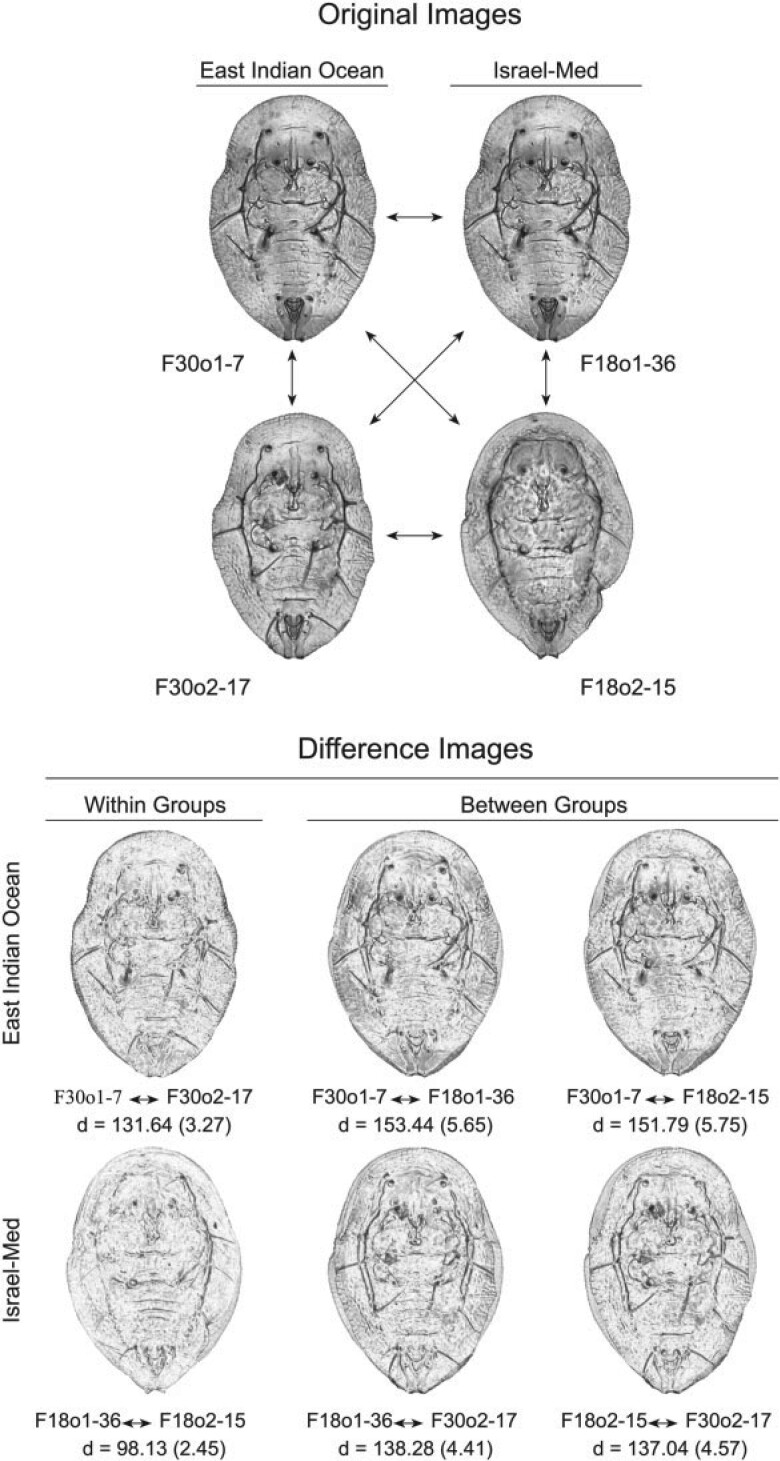
Example of embedded, paired group-contrast comparisons within *Bemisia tabaci* species complex puparial specimens. Upper figure: original images of two randomly selected specimens from the East Pacific Ocean image set (F30o1-7 and F30o2-17) and the Israel-Med image set (F18o1-36 and F18o2-15). Rather than simply comparing image vectors, and so being restricted to small sample sizes, the embedded image comparison protocol used in this investigation focused on pairwise comparisons across all images in the sample (e.g., six comparisons for this four-image example) with the structure of group similarities and differences being represented by Euclidean distances between image vectors. Lower figure: summary of all six comparisons possible for this four-image example in terms of difference images and image distance values ($d)$ for both the full-resolution ($500 \times 500$ pixel) and reduced resolution ($28 \times 28$ pixel) image comparisons, the latter values in parentheses. Here, images that exhibit a higher degree of difference will appear darker than images that exhibit a lower degree of difference, with particular regions and/or structures of distinction being represented as darker areas and/or structures represented by dark highlights. Note that, for both image resolutions, images belonging to the same group typically exhibit shorter image distances relative to those belonging to different groups. Under this approach to the representation of the image (= specimen) similarity structure CNN training focuses on constructing a set of convolution filters that maximize between-groups image distances and minimize within-groups image distances.

One of the most severe limitations of CNN training in many taxonomic and systematic contexts is sample size. Owing to the number of interlayer weights whose values must be calculated recursively, CNNs are usually trained on data sets whose sizes are vast by systematic-research standards. A training set such as ours, consisting of 386 (hairy) and 366 (glabrous) individuals subdivided into 15 classes or groups, would be considered far too small for CNN training by most data scientists. This problem can be circumvented, though, by opting for training as an embedded, distance-based, group-contrast learning system in which the aim is not to learn the characteristics of a priori-defined groups themselves but, rather, implicit patterns of similarities and differences between pairs of images that either do, or do not, belong to the same training group (Fig. 2). Recent published applications of this strategy have focused on systems for describing differences between image pairs drawn from large data sets using text-based descriptors (Jhamtani and Berg-Kirkpatrick 2018; Forbes et al. 2019) as well as image-based analyses (Hall et al. 2014; Hoyal Cuthill et al. 2019; MacLeod and Kolska-Horwitz 2020).

In terms of the analysis of small-to-modestly sized samples, there are many advantages to this approach, including relaxation of the use of single assessments of individual forms insofar as all, or most, pairwise comparisons between images can be employed in training. Thus, despite the fact that our samples contained images of only 386 (hairy) and 366 (glabrous) individuals, totals of 148,610 (hairy) and 133,590 (glabrous) pairwise comparisons can be drawn from them. By focusing CNN training on differences among images of the same group, and between images of different groups, training can proceed more efficiently, and more comprehensively, than would be possible otherwise.

In order to visualize the *B. tabaci* cuticular feature space the t-distributed stochastic neighbor embedding (t-SNE) algorithm (van der Maaten and Hinton 2008; van der Maaten et al. 2009), was employed to summarize the pattern of similarities and differences in puparial morphology in the reduced-dimensional feature space. Again, other dimensionality reduction procedures and algorithms are available (e.g., PCA, see van der Maaten and Hinton 2008; UMAP, see: Bielza et al. 2019; Dorrity et al. 2020). However, as the purpose of this investigation was not to compare the performance of different dimensionality-reduction algorithms on *B. tabacai* data, the t-SNE procedure was selected as an advanced, popular and proven solution to the problem of dimensionality reduction at many different levels of generality.

The t-SNE approach has become a standard dimensionality-reduction technique in many ML contexts and is often now preferred over many longer-established approaches (e.g., PCA, linear discriminant analysis, multidimensional scaling). Owing to its sensitivity, care must be taken when interpreting t-SNE results as it is well known that apparent clustering can result, even in cases where there is no structure (e.g., when applied to data derived artificially from a single statistical distribution, Wattenberg et al. 2016). To avoid this issue, multiple t-SNE analyses were performed using a graded sequence of perplexity and iteration settings. One-thousand—iteration bootstrap variants of Wilk’s $\lambda $ and Pillai’s trace tests (Manly and Alberto 2007) were also used to obtain nonparametric estimates of the statistical significance of the training-set group separations in the trained feature space.

Traditionally, the performance of discriminant functions is tested by evaluating the statistical significance of mean vector separations and by using the trained discrimination system to place members of an independent “validation” set, whose true class identification is known, into inferred groups or classes. Comparative identification accuracies are then typically tabulated in a confusion matrix” Since our sample size was limited, an alternative test of discriminant system stability was implemented via the leave-one-out jackknifed or cross-validation strategy (Manly 2006) which was applied to a randomly selected subset of 40 training-set specimens drawn from the full image sets. For this procedure a sample size of 40 was selected in order to balance the need to base the stability/accuracy test on a representative sample of independent *B. tabaci* puparial images against the time required to train the embedded image LeNet-5 CNN system on a GPU-enabled computer workstation (c. 11 min per cross-tabulation iteration).

## Results

**Figure 3:**
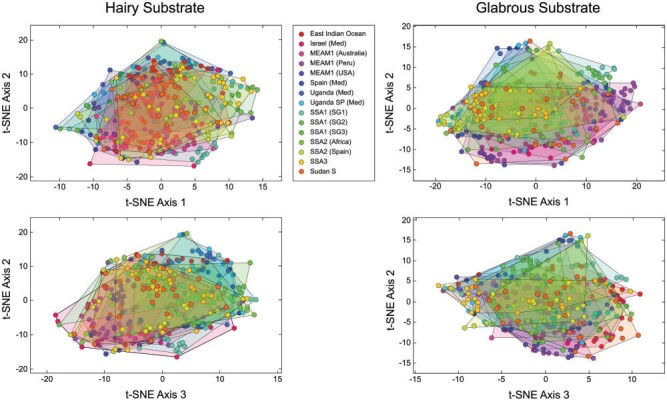
Untrained ordination space for images of the 15 *Bemisia tabaci* groups included in this investigation formed by the first three t-SNE dimensionality-reduction axes. Note lack of obvious between-groups structure. This result suggests that an untargeted assessment of puparial morphology would be insufficient to either identify or quantify between-group morphological differences accurately.

An initial indication of the *B. tabaci* species-level morphological discrimination problem’s magnitude and level of difficulty is illustrated in Figure 3. Here, a quantitative assessment of overall ($=$untrained) levels of between-groups puparial morphological similarities and differences is provided via direct analysis of the 15 sets of *B. tabaci* puparium images using the t-SNE algorithm. The fact that no between-groups structure was revealed by this analysis is consistent with the degree of difficulty experienced taxonomists have reported in their attempts to parse puparial specimen sets into diagnosable groups using classic, qualitative inspection. Owing to the raw, untrained nature of this comparison, however, this result neither demonstrates, nor precludes the possibility that, a more refined comparison between groups, based on morphological data, will fail to uncover such differences. Like all dimensionality-reduction methods, the t-SNE algorithm employs no information regarding group membership in its calculations and so makes no attempt to optimize differences between groups designated a priori.

### Hairy Substrate Specimens

Training of the embedded, group-contrast LeNet-5 CNN system on the hairy-substrate image set consisted of submitting pairwise, Euclidean distance-defined contrasts between the 386 puparial images to the LeNet-5 CNN. The complete training sequence consisted of 1863 iterations of 64 image contrasts each ($=$a batch) with each set of iterations being regard as a training round ($=$an epoch). Training proceeded over ten rounds with each round consisting of 119,196 training-image contrasts. The order of these 119,196 image contrasts was shuffled randomly between rounds and training was allowed to proceed across the entire 10-round (or epoch) cycle. Thus, training was based on the consideration of 1,192,320 pairwise image comparisons despite the fact that only 119,196 unique image contrasts were employed.

Across the ten training rounds performance improved dramatically through the initial rounds and converged to a value very close to 0.0 error approximately midway through the fourth round (see Supplementary Material archive available on Dryad). After this a very low error rate was maintained with small, but prominent, deviations to somewhat increased error rates occurring in a quasiperiodic sequence across the remaining six rounds. These deviations were, however, corrected rapidly by subsequent system training. At the point of training termination, no training-set identification errors were recorded.

Following the training cycle a crude indicator of overall performance was obtained by using the trained system to identify the raw training-set images. Note that this exercise is not quite the same as asking the trained system to identify the images on which it was trained, insofar as the system was not trained on the raw puparial images, but rather on distance-based estimates of contrasts between pairs of puparial images both within and across species groups. This embedded training design assisted the CNN system in its task of focusing training on those aspects of morphological variation most closely associated either with species-group membership ($=$ contrasts between images belonging to the same genetic species) or species-group distinction ($=$ contrasts between images belonging to different genetic species, see Fig. 2). Once trained in this manner, though, the system can be employed to identify raw puparial images. Consequently, it is informative to use the set of raw training images to evaluate trained system performance despite it being true that these same images participated in system training in the sense that they provided the basis for the contrasts on which system was trained.

For our hairy-substrate image set the trained LeNet-5 system was able to identify all of the raw training-set images correctly post hoc (see Supplementary Material archive available on Dryad). The raw accuracy level of identifications for this result is 1.0. However, in the case of confusion matrices with less than perfect identification scores the raw accuracy can be misleading especially when group sizes are not equal. The Matthews correlation coefficient (MCC) is typically used to obtain an accuracy index value that corrects for this source bias (Matthews 1975; Chicco and Jurman 2020). Though its performance has been questioned recently by Zhu (2020), the MCC remains superior both to the raw accuracy index and to coefficients that take only some of the four confusion matrix categories into consideration (e.g., F1 score). Regardless, owing to the fact that our result contained no misidentifications, MCC value for the hairy substrate training-set confusion matrix was also 1.0.

A more rigorous test of trained CNN performance, of course, is to determine how many correct identifications result from submission of a set of previously identified images other than those used to train the system. Unfortunately, sequestration of a reasonable set of *B. tabaci* complex images from the training set for use as a validation set would compromise adequacy of the training set given the already low sample sizes that were available for this investigation. To overcome this problem the cross-tabulation, or jackknife, strategy, involving 40 randomly chosen *B. tabaci* images, was employed.

Despite the fact that the randomly selected subset of 40 validation images did not include specimens from MEAM 1 (Aus), Uganda SP (Med), SS2 (Africa), and Sudan (S), a perfect validation-set identification result was achieved (see Supplementary Material archive available on Dryad). This result addresses any concern that the exemplary results obtained from the post hoc identification of training-set specimens were an artifact of an overtrained discrimination system. It also suggests the LetNet-5 CNN trained on hairy-substrate puparial group contrasts exhibits remarkable stability, implying that it should be capable of highly accurate identifications for (at least) 11 of the 15 groups included in this study. Given these results, it is highly likely that this trained CNN is capable of perfect or near-perfect identification performance for all 15 hairy-substrate *B. tabaci-*complex species groups based entirely on aspects of their puparial morphologies as recorded in light photomicrographs despite the rather simplistic character of the LeNet-5 CNN architecture. The MCC that summarizes the confusion matrix that resulted from this test is, of course, also 1.0.

**Figure 4:**
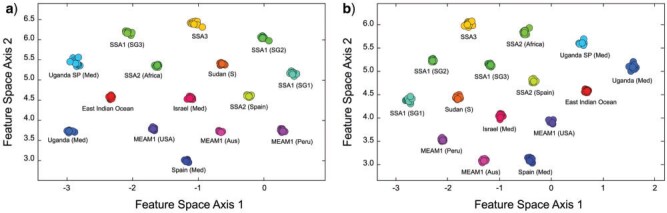
Two-dimensional feature subspace plots of recognized *Bemisia tabaci* genetic/molecular species from the hairy-substrate (a) and glabrous-substrate (b) image sets as delineated by the embedded, group-contrast LeNet-5 CNN based on *B. tabaci* puparial photomicrographs. Note the clear separation of all groups in both image sets indicating the existence of substantial and consistent within-groups morphological similarities and between-groups morphological differences. The extremely tight clustering of species groups, and substantial interspecies gaps, suggest that, even though these species samples might not be fully inclusive of the extreme morphologies present in (some) wild populations, there is ample room to accommodate a substantial degree of intraspecific morphological variation within this discriminant model without necessitating reoptimization.

Owing to our use of a 2D output vector setting, it was possible to create simple graphic illustration of the degree to which the trained, embedded group-contrast LeNet-5 CNN was able to discriminate between these *B. tabaci* species by plotting the 2D feature space of the post hoc training set output-identification values (Fig. 4a). Even within this low-dimensional representation the full *B. tabaci* complex feature space, it is clear the embedded CNN analysis was able both to identify, and to quantify, consistent within-group similarities and between-group differences in these hairy-substrate puparial morphologies with much greater precision than had been delivered by any previous analysis; either quantitative (via morphometrics) or qualitative (via visual inspection by expert taxonomists). Moreover, the fact that taxonomic characterization at this level of detail was extracted from simple, low-resolution ($28 \times 28$ pixel) photomicrographs of puparia suggests that a wealth of useful morphological information resides, not only in these *B. tabaci* specimens but also, by implication, in the morphologies of other organismal groups; information that exists but has remained inaccessible to scientific study until the advent of ML techniques.

Given the level of between-groups distinctions apparent in Figure 4a, statistical tests of group distinction relative to group dispersion seem largely beside-the-point. However, for the sake of completeness and transparency, the bootstrapped Wilks’ $\lambda $ and Pillai trace tests were carried out on these feature-space score data. Both tests rejected the null hypothesis of no between-group differences at very high levels of statistical confidence (Wilk’s $\lambda = 3.10 \times 10^{-7}$, assoc. $F = 4745.0$ with dof: 28, 740; Pillai’s trace $= 2.00$, assoc. $F = 4386.0$ with dof: 28,742).

### Glabrous Substrate Specimens

Since it is well known that *B. tabaci* species’ puparial morphologies change in response to leaf-substrate type, it was necessary to repeat the foregoing hairy-substrate analysis for a set of puparial specimens grown on smooth or glabrous-leafed host plants to achieve a complete test of whether unique morphological distinctions characterize these 15 *B. tabaci*-complex genetic/molecular species, irrespective of leaf form. At 366 specimens, the glabrous-substrate image set was slightly smaller than the hairy-substrate image set, thus yielding a slightly smaller pool of image contrasts for use in CNN training.

Again, batches of 64 images each were iterated 1863 times into a training round ($=$ epoch). Training was allowed to proceed over 10 rounds/epochs with the order in which image contrasts were presented to the system being changed between each cycle. Thus a total of 1,192,320 image contrasts, drawn from a set of 119,196 unique pairwise contrasts, was used to train the glabrous-substrate LeNet-5 CNN system.

As with the hairy-substrate analysis, across the ten training rounds identification performance improved dramatically and, in this case, converged to a value very close to 0.0 error approximately midway through the third training round (see Supplementary Material archive available on Dryad). After this, stability was maintained with small, but prominent, deviations occurring with quasiconstant periodicity as the system attempted to further improve its (already exemplary) performance. Without exception, these deviations were corrected rapidly by subsequent system training. As before, at the end of training on the glabrous substrate specimens set no training-set identification errors were recorded.

Overall performance of the glabrous substrate discriminant system was assessed by using the trained system to identify the raw training-set images post hoc, as a first-stage performance-evaluation exercise. In the case of these glabrous-substrate puparial images the group contrast-trained LeNet-5 CNN system was, again, able to achieve a perfect assignment of training-set puparial specimens to their correct species based on the morphological data recorded in their transmitted-light photomicrographs (MCC $= 1.0$; see Supplementary Material archive available on Dryad).

These results may be surprising to readers who have experience with multivariate linear discriminant analysis as well as, perhaps, a few with prior ML analysis experience. But, again, it must be stressed that LeNet-5 CNN was trained on between-species image contrasts rather than on the raw images directly. As such, these glabrous-substrate results suggest, at the very least, that the trained CNN should be able to provide reliable and accurate—as well as very rapid—identifications of all 15 glabrous substrate-reared *B. tabaci*-complex genetic/molecular species’ puparia on the basis of morphological data alone.

A more rigorous test of the performance and stability of LeNet-5 CNN trained on glabrous-substrate *B. tabaci* puparial images involved the same cross-tabulation, or jackknife, design employed during evaluation of the hairy-substrate LeNet-5 discriminant system. Once again, 40 randomly selected validation-specimen images were successively sequestered from the remaining 365 images which were then used to retrain the embedded, group-contrast LeNet-5 CNN system. In this case, specimens from all but one (Uganda [Med]) of the training-set genetic/molecular species were part of the random cohort selected for validation analysis. Nonetheless, for each of these included species—some of which were represented by as many as eight specimens—a perfect set of cross-tabulation identifications were obtained (MCC $=$ 1.0). This result effectively rules out any concern that the exemplary results obtained from the post hoc identification of glabrous-reared training-set specimens were an artifact of an overtrained discrimination system.

Our level of confidence in this interpretation does not rest solely on the results of these cross-tabulation tests, which measure the ability of the trained CNN systems to be assigned to correct groups based on their linear proximity to group centroids (the typical decision criterion for evaluating quantitative discriminant-system performance). Indeed, it is often the case that exceedingly good apparent levels of discriminant performance can be returned, even from data sets whose extremes of group-level variation overlap quite strongly. Rather, confidence in our results, and in the ability of ML methods generally, to deliver results that approximate those of the Gestalt taxonomist’s concept of species differences, lies in a combination of these results and in the character of the feature space from which our cross-tabulation results have, ultimately, been derived.

Figure 4b shows the estimated 2D feature space for the 15 *B. tabaci* complex species reared on glabrous leaf substrates. Obviously, despite its simple architecture, the trained LeNet-5 CNN was successful in finding morphological features among these *B. tabaci*-complex glabrous substrate-reared groups that exhibited exemplary intra-group consistencies as well as rather dramatic between-groups differences. Close comparison with Figure 4a shows that, if anything, this feature-space exhibits slightly more dramatic group-level distinctions than those obtained for the hairy substrate-reared puparial images. Logically this result also seems consistent insofar as the more complex hairy substrate leaves would be expected to increase the degree of morphological response in puparial form. Despite this very minor, and qualitatively judged, difference between the two analyses though, the character of the morphological distinctions exhibited by glabrous substrate-reared specimens was not more significant statistically. Both the Wilks’ ($\lambda_{\rm wilks}$) and Pillai trace ($\lambda_{\rm pillai}$) tests were carried out on these glabrous-substrate feature-space score data and both tests rejected the null hypothesis of no between-group differences at very high levels of statistical confidence (Wilk’s $\lambda = 0.022$, assoc. $F = 143.0$ with dof: 30,738; Pillai’s Trace $= 1.67$, assoc. $F = 124.8$ with dof: 30,740).

### Comparison of Results

Owing to the fact that CNN training relies on random selection of batches of training-set images, it is expected that different training cycles will yield slightly different results, even for the same set of images. Thus, at this initial stage of our work with these genetic species, we are reluctant to offer a confident interpretation that the greater degree of intra-specific variability our results show for the hairy substrate-reared sample reflects a genuine biological difference rather than an idiosyncrasy of these two CNN training cycles. Resolution of this issue must await further investigation.

Similarly, in order for these results to be considered useful in systematic and/or outbreak-mitigation contexts, it must be the case that the specimens included in our sample were fully representative of their corresponding wild *B. tabaci* species complex populations. Since our specimens were reared under strict laboratory conditions (see Materials and Methods section), this requirement cannot be validated empirically. Moreover, given the rather modest sizes of some species’ samples we cannot guarantee that all of the more extreme specimen morphologies present in wild populations would have been included in our samples. Our sample sizes, however, are sufficient to have recorded accurate estimates of mean and close-to-mean species’ morphologies, which would be expected to be characteristic of the greater proportion of any wild population.

Irrespective of these considerations, given the strength of our cross-tabulation results, we feel confident in claiming that, on the basis of the evidence we have provided, the LeNet-5 system trained using the embedded, group-contrast protocol was able to deliver highly accurate identification results for typical *B. tabaci*-complex puparial specimens across any of the 15 valid species included in our investigation based on puparial morphology alone, irrespective of whether the puparia were grown on hairy or glabrous leaf substrates. Future investigations may show it is possible to train a ML system to identify *B. tabaci* specimens irrespective of which substrate they were grown on. For now though, there can be little doubt that i) broad and consistent morphological distinctions do appear to exist between these *B. tabaci* complex genetic/molecular species irrespective of the substrate on which they were grown, ii) these distinctions are sufficiently prominent to make the automated identification of species belonging to this complex from their pupal morphologies a practical possibility, at least in principle and iii) such an ability, if confirmed by additional research, could not only have a substantial impact on research efforts directed at understanding *B. tabaci* biology, but also in efforts to control and/or (perhaps) eradicate outbreaks of these commercially damaging agricultural pests. In addition, results obtained by this investigation have demonstrated the power of advanced ML approaches to the investigation of morphological variation and, indeed, how much interesting and useful, but heretofore unsuspected, information is likely to be present in biological morphologies generally.

## Discussion

Molecular analysis and DNA barcoding studies are clearly important, relatively inexpensive and easily accessed tools for taxonomy (Hebert et al. 2003; Vogler and Monaghan 2007); especially useful for diagnosis and delimitation, and in the discovery of cryptic species (Tan et al. 2009; Dinsdale et al. 2010; Lee et al. 2011). However, despite the insight provided by taxonomy-focused molecular research, many practical problems remain. For example, the relatively rapid rate at which new cryptic species continue to be discovered has led to many changes in the use of informal names, creating confusion within the scientific literature (Boykin et al. 2018). This nomenclatural confusion also interferes with classification and biodiversity assessments (Tan et al. 2009). Complications inherent in molecular barcode data can also exacerbate these problems (see Wright et al. 2006; Vyskočilová et al. 2018).

Additionally, molecular divergence thresholds used for species delimitation are arbitrary (DeSalle et al. 2005; Meier et al. 2006) and are often computed incorrectly (Meier et al. 2008). Such thresholds vary widely across taxa (e.g., Britten 1986; Sanderson 2002; Smith and Donoghue 2008; Lee et al. 2013) primarily due to variation in the substitution rates associated with species-specific molecular clocks (Wright et al. 2006; Bromham 2009). Genetic divergence within the *B. tabaci* complex has been shown to be higher than the interspecific genetic divergence of related genera (Lee et al. 2013). This has led to the 3.5$\%$ mtCO1 region threshold—used originally in *B. tabaci* species delimitation (Dinsdale et al. 2010)—to be regarded as erroneous by Lee et al. (2011) and Vyskočilová et al. (2018). Lee et al. (2011) estimated the correct threshold to be 4.0$\%$, although this limit appears not to have been adopted in some subsequent studies (e.g., Vyskočilová 2019). The work of Kanakala and Ghanim 2019), which did employ the 4.0$\%$ threshold, recognized 44 putative *B. tabaci* species. In addition, the presence of pseudogenes can result in novel species being reported erroneously. Delatte et al. (2007) proposed a new *B. tabaci* molecular species, MEAM2, which was later shown to be a pseudogene artifact (Tay et al. 2017). Consequently, it seems likely other unidentified pseudogenes exist within the *B. tabaci* complex.

Given these issues, coupled with the fact that most biological species have been defined on the basis of morphological criteria, we agree with Hillis (1987), Dayrat (2005), Tan et al. (2009), and Schlick-Steiner et al. (2010), Palandaéiæ et al. (2017), Dzhembekova et al. (2020), and others that species identifications should strive to integrate data from different sources rather than preferencing one source over others. Our preliminary research on the *B. tabaci* complex molecular/genetic suggests how this might be done in an effective and practical manner.

While various aspects of *B. tabaci* biology suggested it might be a cryptic species complex, widely accepted proof that such was indeed the case required use of DNA sequencing (Dinsdale et al. 2010; Kanakala and Ghanim 2019; Vyskočilová 2019). However, the full extent of biological differentiation among *B. tabaci* species cannot be assessed from DNA-sequence data alone. All previous attempts to ascertain the extent of morphological differentiation among *B. tabaci* species defined (provisionally) by genetic/molecular criteria failed to identity morphological distinctions between any, or all but a few, genetic species (Li et al. 2013; Chaubey and Andrew 2015; Harish et al. 2016). These failures have been interpreted to mean that there were no consistent morphological distinctions between *B. tabaci* species recognized on the basis of genetic/molecular criteria. As our results have shown, this interpretation was the product of limitations in the methods employed to assess distinctions between *B. tabaci* purparial images, but not of *B. tabacai* morphology itself.

At least 15 *B. tabaci* species defined (provisionally) by genetic/molecular criteria also display consistent patterns of morphological variation in the forms of their puparia. These patterns were not recognized by previous morphometric investigations because they are not reflected in the gross dimensions of the puparial outline and because there are an insufficient number of stable, relocatable landmark positions available to characterize the forms of these complex biological objects to the degree of detail necessary to identify species-specific morphological differences. This is not an uncommon situation in morphometric analyses. Nonetheless, when whole images of genetic/molecular delineated *B. tabaci* puparial specimens were compared with one another using even the simplest and most straightforward of CNN system architectures, the existence of distinct, consistent and stable differences in the puparial forms of these putative species was revealed. Moreover, the most recent study of *B. tabaci* phylogeny, using whole-genome and mating compatibility data, failed to fully delimit the SSA1-SG1, SSA1-SG2, and SSA1-SG3 taxa (Mugerwa et al. 2020). Yet, our group-contrast trained LeNet-5 CNN system demonstrated the existence of both clear and stable morphological differences between these three taxa, thus providing evidence that at least three additional *B. tabaci* species may exist within the SSA1 group.

Given the outstanding quality of the species-discrimination results we obtained for both hairy and glabrous substrate-reared puparial morphologies, it seems natural to inquire what the morphological bases of the distinctions identified by the group-contrast trained LeNet-5 CNN systems might be. Unfortunately, gaining insight into the variables, features or regions of an image (or other data) that prompts assignment to a particular group category is not a straightforward a matter for advanced ML systems (Molnar 2020). This is by no means unusual in systematic investigations. Any number of cluster-analysis results have been published in the systematic literature with scant attention paid to the quantitative investigation, or even the qualitative interpretation, of the respective contribution(s) of the original variables to the cluster-pattern result. Nevertheless, this is an active area of ML research that, in time, promises to contribute much to our understanding of the morphological discontinuities systematists observe in nature.

Permutation feature importance (PFI, Breiman 2001), saliency maps (Simonyan et al. 2014), local interpretable model-agnostic explanations (LIME, Ribeiro et al. 2016), Shapley values (Shapley 1951; Lundberg and Lee 2017), scoped rules or anchors (Ribeiro et al. 2018; Delaunay et al. 2020) and neural-backed decision trees (NBDT, Wan et al. 2020) are among the procedures available presently to evaluate the targets of inter-group ML discriminations. To date, such approaches have not been able to provide levels of spatial detail sufficient to identify the critical characters character states, and/or geometric trends responsible for group differences (see Arteaga 2019; Stewart 2020 for examples). But the potential of these (and other, similar) procedures is clear and improved variations of these data-analysis themes are being developed. Presently, these approaches can be useful in ensuring group-diagnostic image features belong to parts of the image that pertain directly to the specimens being imaged (e.g., as opposed to some aspect of the background). In the case of our *B. tabaci* species-complex analyses we were careful to remove all aspects of the images in question beyond the specimen’s peripheries, so there could be no question of our system’s identifications being based on nonspecimen-based morphological data.

Another issue of potential concern is the apparent absence of strict conformance to topological homology relations, such as those maintained characteristically in landmark-based GM analyses (see Bookstein 1991). This potential avenue of criticism is both true and false. The Cartesian representation of form is explicit in its use of landmark location-coordinate values whereas, in the case of digital images, it is implicit in the configuration of pixels (akin to semilandmarks) within a pixel frame. Both of these representational strategies are consistent with configurations of coordinates in Kendall’s (1981, 1984) shape space. But in the case of image-pixel data, such configurations encode more complete and detailed sets of morphological descriptors and can include other sorts of useful information (e.g., color values).

Perhaps more importantly, data consisting of sparse sets of landmark points, by their very nature, will always be restricted to the characterization of a small subset of the morphological features present in complex organic structures, and restricted to those structures that are similar in form as well as being able to be located on all specimens within a sample. In contrast, image-pixel data sets, by their very nature, are sensitive to differences among all features present in the image frame. The comprehensiveness of this representation is the primary advantage of using image-pixel data to quantify patterns of morphological variation. This is why the analysis of digital images, as opposed to sparse sets of landmark locations derived from such images, are the subject of most ML and computer vision applications irrespective of the fact that it is both quick and easy to reduce even complex images to sets of key point locations and match these across image sets using automated algorithms (see Harris and Stephens 1988; Sánchez et al. 2018).

But irrespective of such considerations, quantitative and traditional qualitative approaches to the analysis of morphological data and, indeed molecular/genetic assessments of population divergence, should not be seen as competitors for the systematist’s attention. All can, and should, be employed in a reciprocal manner to explore differences among biological species and so contribute to the scientific understanding of their character. Owing to the costly damage *B. tabaci* infestations inflict on agricultural crops, this understanding can, and should, be extended to devising ever more effective strategies for their control. There is great potential in combining expert visual inspection, genetic/molecular-validation, GM and ML-based strategies in the context of quantitative morphological investigations, and strong suspicion that doing so will only enhance the already impressive “stand-alone” powers of each.

Finally, we’d like to offer a closing recommendation regarding the systematics of *B. tabaci*. Given the weight of the evidence in favor of between-species morphological distinctions, along with the economic importance of a number of *B. tabaci*. complex species, we believe the taxonomic community is now in a position to proceed with the formal description of at least the species known most commonly as “SSA1” (the cassava pest) as a matter of some urgency. Taking this action will not only serve as an important signal of the true nature of this species group, it will encourage other researchers to begin the task of systematic revision in light of the information we now possess in the areas of molecular systematics, genetics, phylogenetics, biogeography, ecology and, most recently, morphology. Other species in the complex may be described either as new, or identified as existing species, e.g., it has been established that the species referred to as “Med” or “Q-biotype” represents *B. tabaci* sensu stricto (Tay et al. 2012).

## Conclusions

Our morphology-based results i) offer support for existing evidence that at least 15 *B. tabaci* complex genetic/molecular species groups do represent valid biological species, ii) provide an alternative (and possibly more efficient) means of identifying the source(s) of new *B. tabaci* outbreaks for these 15 genetic/molecular species, and so contribute to the mounting of maximally effective eradication, mitigation and/or control procedures, and iii) place future investigations into the developmental, ecological, biogeographic and phylogenetic aspects of this complex’s biology on a much firmer taxonomic footing. Now that morphological differences have been demonstrated to exist among genetic/molecular species included in the *B. tabaci* complex, use of ML techniques can be expanded to include other genetic/molecular species within this, and other, species complexes. Additionally, work can now begin on identifying the specific morphological differences that characterize these *B. tabaci* species uniquely.

In the same way mathematics is regarded by many mathematicians as the study of patterns in numbers (Hardy 1940), systematic biology can be thought of as the search for patterns in the living world, both in time and space. It should never be forgotten that it is the existence of such patterns that provides the subject matter for all biological studies as well as the evidence that deterministic processes or factors are responsible for their creation. If such patterns did not exist—if everything in the living world simply graded continuously and insensibly into everything else—it would not only be impossible to conduct any truly scientific biological investigation, such studies would be pointless.

Mathematical data analysis, statistics and machine learning are tools that, when employed properly, can be used to discover and document patterns in natural history data that can aid systematists in their investigations and hypothesis tests. They are not, substitutions for, or means through which, careful reasoning by researchers with specialist knowledge and experience can be overruled. Rather they can, are, and should be, used to aid and support biological reasoning by extending the power of human senses and perception; by making patterns invisible to the unaided eye visible so they can be identified, discussed and interpreted.

Getting from the data to the interpretations via analysis and reasoning is a primary challenge of biological research. While mathematics can be of great assistance in attaining this goal—especially the newer forms of image-based ML and artificial intelligence—there is not, and never will be any, easy, straightforward, and infallible way to accomplish this aim. Nevertheless, the fact that the use of quantitative data-analysis strategies is neither simple, straightforward, nor infallible is no reason to abjure their employment in wider biological research contexts generally, and in wider systematic and taxonomic contexts in particular, than they enjoy presently. Most importantly, the development of new and ever more sophisticated ways of applying quantitative data-analysis procedures to identify patterns in biological data promises to invigorate, and perhaps to revolutionize, systematic biology by providing access to patterns in the morphological domain (as well as in other domains) of which, to this point, we have scarcely been aware.

## Supplementary Material

Data available from the Dryad Digital Repository: http://dx.doi.org/10.5061/dryad.sqv9s4n39.
